# Thyroiditis mimicking relapse of acute lymphoblastic leukemia: Gallium-67 scan suggested the diagnosis

**DOI:** 10.4103/0971-5851.73601

**Published:** 2010

**Authors:** Saleh Othman

**Affiliations:** *Department of Radiology and Medical Imaging, Nuclear Medicine, King Khalid University Hospital and College Of Medicine, King Saud University, Riyadh, Saudi Arabia*

**Keywords:** *Acute lymphoblastic leukemia*, *gallium-67 scan*, *thyroiditis*

## Abstract

Acute lymphoblastic leukemia (ALL) is the most common form of leukemia in childhood and accounts for 85% of cases. ALL frequently presents as an infectious process with an abrupt onset of high fever. Thyroid disease has been reported to have a strong association with acute leukemia. Gallium (Ga-67) citrate has been used in the investigation of patients labeled as having pyrexia of unknown origin. We report a case of a 13-year-old female patient who presented with fever and suspected disease relapse after a period of disease remission; however, gallium-67 citrate whole body scan suggested the diagnosis of thyroiditis.

## CASE REPORT

**Figure 1:**
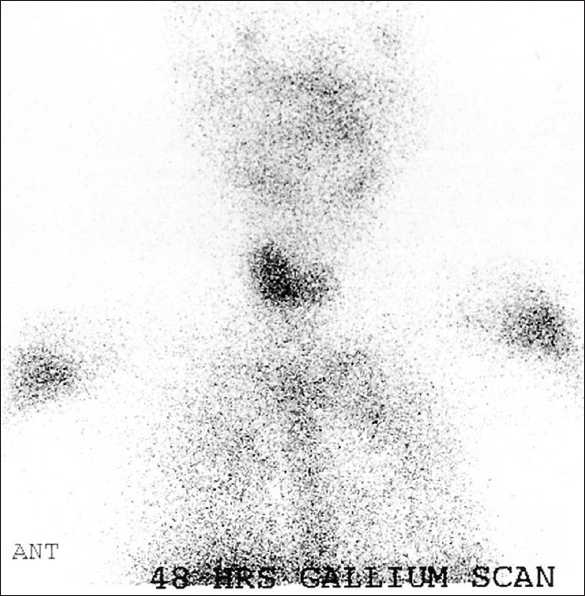
48 hours Ga-67 citrate (spot image of the neck) showing enlarged thyroid lobes with increased tracer uptake (right > left)

**Figure 2:**
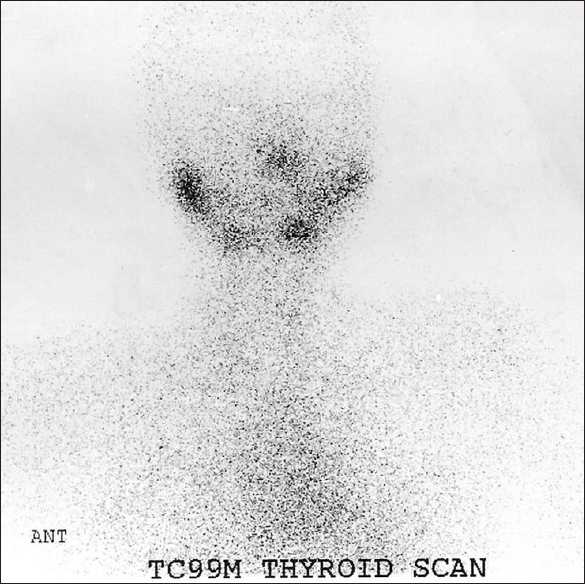
Tc-99m pertechnetate thyroid scan did not show any tracer concentration by the thyroid gland

A 13-year-old female patient, a known case of acute lymphoblastic leukemia (ALL), received chemotherapy and was in remission for more than 1 year. She presented with fever, tachycardia, and sweating. She was admitted for investigation for possible relapse. Among the imaging procedures ordered was gallium-67 citrate whole body scan. Total body gallium scan was normal except for the prominent increased uptake in the enlarged thyroid gland [Figure 1], suggesting an inflammatory process (e.g. thyroiditis). Thyroid scan was performed using technetium 99m and showed no tracer uptake by the thyroid gland [Figure 2], raising the probability of thyroiditis suspected on gallium scan. Thyroid function test showed raised FT4 and suppressed thyroid stimulating hormone (TSH). All these findings explained the signs and symptoms of the patient, which were related to thyrotoxicosis and not to relapse of her original disease.

## DISCUSSION

Leukemia is the most common childhood malignancy and accounts for 30–40% of all malignancies. ALL is the most common form and accounts for 85% of cases.[1] ALL frequently presents as an infectious process with an abrupt onset of high fever. Several chemotherapy regimens have been used to treat ALL and remission of more than 3 years for most patients has been reported. However, after cessation of chemotherapy, the relapse rate is about 15% and most occur within the first year.[2] The relapse can also present with an episode of fever. Association of thyroid disease with acute leukemia is well known and has been reported by many authors.[3–4] Also, it has been reported that there is a strong association of autoimmune thyroid disease and acute leukemia, and autoimmune thyroiditis can be manifested as a systemic febrile illness.[5] Isotope imaging has been used in inflammatory thyroid disorders.[6] Thyroid uptake of Ga-67 has been reported in patients undergoing investigations for systemic febrile illness.[7] Achong et al. reported intense Ga-67 accumulation by the thyroid gland in a man with AIDS, imaged for suspected Pneumocystis carinii pneumonia. Concurrent Tc-99m pertechnetate thyroid scanning demonstrated absent trapping, helping to establish the diagnosis of painless thyroiditis.[8]

In summary, in patients with treated ALL, who are in remission and present with an episode of fever together with other symptoms suggestive of disease relapse, the probability of thyroiditis should be considered and gallium scan may aid in confirming the diagnosis.

## References

[1] Bernard EJ, Nicholls WD, Howman-Giles RB, Kellie SJ, Uren RF (1998). Patterns of abnormality on bone scans in acute childhood leukemia. J Nucl Med.

[2] Berkow R (1982). The acute leukaemias. The Merck Manual.

[3] Willems E, Valdes-Socin H, Betea D, Beckers A, Beguin Y (2003). Association of acute leukemia and autoimmune polyendocrine syndrome in two kindreds. Leukemia.

[4] Byrd JC, Dow NS, Gaertner E, Hargis JB, Raber TR, Burrell L (1997). Leukemic thyroiditis as the initial relapsing sign in a patient with acute lymphocytic leukemia and blast expression of the neural cell adhesion molecule. Am J Hematol.

[5] Moskowitz C, Dutcher JP, Wiernik PH (1992). Association of thyroid disease with acute leukemia. Am J Hematol.

[6] Matthies A, Nikpoor N (1997). Isotope imaging in inflammatory thyroid disorders.

[7] Castellucci RP, Gardner DF, Haden HT, Adler RA (1989). Autoimmune thyroiditis manifested as a systemic febrile illness: Diagnosis by gallium scan and fine needle aspiration biopsy. South Med J.

[8] Achong DM, Snow KJ (1994). Gallium-avid painless thyroiditis in a patient with AIDS. Clin Nucl Med.

